# Biophysical properties and antimicrobial efficacy of a novel Silk Sericin Mineral Trioxide Aggregate

**DOI:** 10.3389/fdmed.2025.1615724

**Published:** 2026-01-05

**Authors:** Raghavendra M. Shetty, Shyam Kumar Vootla, Nilima Thosar, Tarun Walia, Vijay Desai, Anas Al Jaada, Elias Berdouses, Kusai Baroudi, Anup Hemant Eden, Apurva Mishra, Sunaina Shetty Yadadi

**Affiliations:** 1 Department of Clinical Sciences, College of Dentistry, Ajman University, Ajman, United Arab Emirates; 2 Center of Medical and Bio-allied Health Sciences Research, Ajman University, Ajman, United Arab Emirates; 3 Department of Pediatric and Preventive Dentistry, Sharad Pawar Dental College and Hospital, Wardha, Maharashtra, India; 4 Department of Biotechnology and Microbiology, Karnatak University, Dharwad, Karnataka, India; 5 Department of Restorative Dentistry, College of Dental Medicine, University of Sharjah, Sharjah, United Arab Emirates; 6 Microbiota Research Group, Research Institute of Medical and Health Sciences, University of Sharjah, Sharjah, United Arab Emirates

**Keywords:** biomaterials, endodontic procedures, pulpotomies, silk protein, sericin, sericin-MTA, MTA

## Abstract

**Background:**

Mineral Trioxide Aggregate (MTA) is widely used in endodontics due to its excellent biocompatibility and sealing abilities. However, complete eradication of bacteria from the root canal remains challenging. Currently available materials cannot ensure a complete hermetic seal and antimicrobial properties, which are essential to prevent reinfection. This study aimed to develop a novel Mineral Trioxide Aggregate combined with silk sericin and to evaluate its biophysical properties and antimicrobial efficacy against oral pathogens.

**Methods:**

Lyophilized silk sericin powder was mixed with MTA in a 1:1 ratio. Silk sericin-MTA Conjugate (SC), Silk sericin (SS), and MTA were evaluated for their biophysical properties using Attenuated total reflectance-Fourier transform infrared (ATR-FTIR) spectroscopy, x-Ray diffraction, atomic force microscopy, and circular dichroism. The antimicrobial efficacy was evaluated against Staphylococcus *aureus*, Enterococcus *faecalis*, and Candida *albicans* using MIC, MTT, and time kill assay.

**Results:**

ATR-FTIR spectroscopy revealed distinctive peaks, confirming the structural modifications of the Sericin-MTA conjugate. x-ray diffraction revealed that all groups exhibited a lattice structure and were found to be crystalline in nature. However, in atomic force microscopy, MTA appeared to have a flat and uniform surface, while Sericin-MTA conjugate showed shallow depressions across its surface. The circular dichroism revealed the typical properties of silk protein, which is mainly composed of α-helical structures in Sericin-MTA conjugate. The antimicrobial activity was observed in the order of MTA < SC < SS.

**Conclusions:**

This research provides concrete evidence that the innovative combination of MTA and silk protein enhances antimicrobial efficacy, making it a promising biomaterial for use as a medicament in endodontic procedures.

## 1 Introduction

Mineral Trioxide Aggregate (MTA) is a substance of non-biological origin that has been used in dentistry for more than two decades for a variety of procedures in restorative dentistry and endodontics. MTA is a tricalcium silicate cement that supports osteogenesis and healing because of its hydrophilicity and biocompatibility (1). It is a mixture of various trioxides, including silicon oxide, tricalcium oxide, and bismuth oxide, as well as several hydrophilic particles, such as tricalcium aluminate and tricalcium silicate. Each of these components contributes to its physical and chemical properties. In the presence of moisture, these hydrophilic particles facilitate the hardening of MTA (2–5). This hydration reaction leads to the formation of a colloidal gel, resulting in a structure that solidifies in about 4 h with a 12.5 pH (6–9).

The majority of endodontic failures are the result of inadequate bacterial elimination from the root canal (10). Therefore, in addition to providing an effective sealing ability, it is imperative for a material to have some antimicrobial activity to inhibit bacterial growth post-treatment (11). An overview of the literature reveals that MTA exhibits limited antibacterial activity (12–14). MTA has been extensively used as a retrograde filling material. However, the spaces between a retrograde cavity and restoration create pathways for bacterial products to penetrate the root canal system, leading to the failure of a hermetic seal and the restorative procedure (15).

In the last two decades, silk has advanced from its traditional textile use to biological applications. As a suture material, silk has been extensively used due to its properties such as biocompatibility, non-toxicity, non-irritating, and excellent mechanical properties (16). Based on their physicochemical and biological characteristics, silk proteins such as sericin and fibroin have been utilized extensively in a variety of biomedical applications, such as dentistry, wound dressings, and drug delivery systems (17); textile-based therapy for atopic dermatitis (18); photocatalytic silk mask paper (19); silk-based tissue engineering (20); skin grafts and artificial skin (21); bone grafts (6); artificial ligament and tendon (7); cardiac tissue (8); liver modules (9); artificial pancreas (22); artificial intervertebral disc (23); silk sutures (24); drug delivery (25); protective clothing (26); optics and sensing (27, 28).

Silk sericin (SS) is a biomaterial derived from silkworm cocoons that is used in many wound-healing processes. It has been modified and combined with other materials to expand its range of biomedical applications (29–32). Silk sericin exhibits healing, moisturizing, antioxidant, antimicrobial, anti-ultraviolet radiation, and antitumor characteristics (30, 33). The incorporation of 5 wt.% silver fibroin solution in MTA demonstrated improved handling, ease of manipulation, reduced setting time, and strong diametral tensile strength (34, 35). Given these complementary characteristics, combining sericin with MTA represents a rational and innovative approach to overcoming the inherent limitations of MTA. Such a composite biomaterial has the potential to provide enhanced antimicrobial efficacy against endodontic pathogens, improved handling properties, and better clinical outcomes.

Therefore, the present study aims to develop a novel biomaterial by extracting the silk protein sericin and incorporating it into the conventional MTA with the objective of enhancing the biophysical properties and antimicrobial efficacy of MTA against oral pathogens.

## 2 Materials and methods

Ethical approval was obtained from the Ajman University Research Committee (Ref No.: D-F-H-11-Oct).

### 2.1 Preparation of samples

#### 2.1.1 Silk sericin preparation (SS)

Silkworm *Bombyx mori* cocoons were purchased from a state government cocoon market in Dharwad, Karnataka, India. High-quality cocoons were sorted and used for the extraction of silk proteins. Briefly, cocoon shells were uniformly cut into 1 cm size, and silk sericin protein was hydro-extracted using 0.02% Na_2_CO_3_ and a reflux condenser for 1 h. Hydrophilic dissolved sericin was loaded into a dialysis tube of pore size 6–8 kDa (Spectrumlabs.com) and dialyzed against deionized water for a duration of 3 days. This water was initially changed at intervals of 1 h, 3 h, and 4 h, followed by twice a day. After dialysis, the dialysate was freeze-dried to obtain water-soluble sericin powder, which was used for further experiments. Freeze drying was done using ANM Freeze Drier FD-50-B at a condenser temperature of −80 °C a temperature of sample around −25 °C, and a vacuum pressure of 1 Pa. 100 mg of freeze-dried sericin was mixed with 100 mg of MTA without adding water, and this was done at 25 °C and stored in a vacuum desiccator for further use.

#### 2.1.2 Mineral trioxide aggregates (MTA)

ProRoot MTA white (Dentsply Sirona, OK, USA) was purchased from local distributors in the United Arab Emirates. ProRoot MTA white consists of tricalcium silicate (51.9%), dicalcium silicate (23.2%), calcium di aluminate (3.8%), bismuth oxide (19.8%), and calcium sulfate dehydrated (1.3%).

#### 2.1.3 Silk sericin MTA conjugate preparation (SC)

Lyophilized silk sericin powder was mixed with MTA in a 1:1 ratio based on earlier studies in which the concentration of MTA/sericin was kept 50% of the total composite (36, 37). 50 µl of sterile water was added for a 0.5 g sample, made into a paste, and allowed to dry until further use. All the samples were stored in a vacuum desiccator for 48 h prior to the experiment.

### 2.2 Biophysical characterization of MTA sericin samples

#### 2.2.1 Attenuated total reflectance-fourier transform infrared (ATR-FTIR) spectroscopy

FTIR spectra of the SS, MTA, and SC were obtained using an FTIR spectrometer (Smartitx Thermo Scientific MODEL; Nicolet iZ10) with spectral resolution from 4,000 to 400 cm^−1^ at room temperature.

#### 2.2.2 X-ray diffraction

To investigate the crystallinity of the samples, a conjugate by XRD was done (x-ray Diffractometer-Pwder Model-Smartlab SE) with Cu-Ka radiation (*λ*1–5,405,980 nm) and 45 kV and 40 mA voltages and current, respectively, were used in continuous mode. The scan range was 5–70° with a scan speed of 2°2 per min and a step size of 2*θ* 0.001.

#### 2.2.3 Atomic force microscopy

The sample was evenly placed on a glass slide and was analyzed with a dynamic force mode atomic force microscope (Nanosurf, Flex AFM). The spring cantilever's length of 125 μm, width of 40 μm, and thickness of 2 μm were set. The surfaces were analyzed at room temperature, and 256 × 256-pixel resolution images were obtained. The images were analyzed with image processing and analysis software to measure surface roughness.

#### 2.2.4 Circular dichroism

A widely used application of CD spectroscopy is identifying structural aspects of proteins and DNA. The peptide bonds in proteins are optically active and the ellipticity they exhibit changes based on the local conformation of the molecule. Analysis was done using a Jasco (J-1500) CD Spectrophotometer.

#### 2.2.5 Antimicrobial assay

The agar well diffusion method was used for the microbial assay. Sericin was examined for antimicrobial properties using three test organisms: *Staphylococcus aureus, Enterococcus faecalis, and Candida albicans.* The agar plate surface was inoculated by spreading a known volume (100 µl) of the microbial inoculum over the agar surface. Then, a hole with a diameter of 6–8 mm was punched aseptically with a sterile cork borer. A volume (50 µl) of the antimicrobial agent at the desired concentration of 0.5 mg and 1 mg/50 µl was added to the well. Agar plates were incubated at 37 °C under suitable conditions depending upon the test microorganism. The antimicrobial agent diffuses into the agar medium and inhibits the growth of the microbial strain tested. The activity was performed with sericin (SS), sericin MTA conjugate (1:1) (SC), and a concentration of ProRoot MTA 4–5 mg/50 µl was used.

#### 2.2.6 Minimum inhibitory concentration assay

In the current investigation, test substances were screened using a range-finding test, which measures anti-microbial activity at concentrations between 10 µg and 100 µg/ml. The test compounds were examined against S*. aureus*, *E. faecalis*, and *C. albicans* by a micro-broth dilution assay technique. In brief, microbial cultures containing different concentrations of test samples for each strain were loaded into polystyrene-sterile flat-bottom 96-well plates. The starting inoculum for each strain was 1.5 × 10^5^ CFU/ml, and the wells containing microbial inoculum without any test compounds were used as controls. The plates were incubated at 37 °C. The absorbance of microbial cultures with test samples was measured using a microplate reader. The MIC activity of the test samples was evaluated based on the absorbance. The lowest concentration of compounds that showed neither visible bacterial growth nor turbidity after 24 h of incubation in a micro-broth dilution assay was considered the MIC.

#### 2.2.7 MTT assay

The MTT method was used to study the effects of SS, MTA, and SM conjugate. Control samples included culture medium alone and medium with bacteria but without test compounds. The plates were incubated at 28 °C for 9 h. 5 mg/ml MTT [3-(4,5-dimethylthiazol-2-yl)-2,5-diphenyltetrazolium bromide] solution was prepared in PBS (pH 7.2). Each well received 20 µl of the MTT solution, and the 96-well microtiter plates were incubated for 30 min at 28 °C. After incubation, 80% of the MTT solution was carefully removed to preserve the formazan crystals. The insoluble purple formazan granules were then dissolved using an MTT lysis buffer containing 0.5% Sodium dodecyl sulphate, 36 mM HCl, and isopropanol acid. Absorbance was measured at 560 nm by an ELISA plate reader (Biobase Biodustry-EL10). The final absorbance for each well was calculated as: *Absorbance (560 nm) of the sample—Absorbance (560 nm) of the control*.

#### 2.2.8 Time kill assay

Antimicrobial assay was performed in glass tubes containing 10 ml of Mueller-Hinton broth. The test samples were used at their MIC concentrations. An inoculum containing approximately 5 × 10^5^ cfu/ml was introduced into the Mueller-Hinton broth containing various test samples and incubated at 37 °C. A 500 μl sample was removed from culture at 2, 4, 8, 16, and 24 h, 100 μl of the samples were inoculated on Mueller Hinton agar and incubated at 37°C for 24 h. Control included extract-free Mueller Hinton broth seeded with the test inoculums, viable counts were calculated to give CFU/ml, and kill curves were plotted with time against the logarithm of the viable count. Further, the results of the experiment were studied using the following parameters:

$$\text{\%}\mathrm{reduction}=\frac{\mathrm{Initial}\;\mathrm{count}\;\mathrm{without}\;\mathrm{drug}\text{-}\mathrm{cell}\;\mathrm{counts}\;\mathrm{after}\;\mathrm{incubation}\;\mathrm{with}\;\mathrm{drug}}{\mathrm{Initial}\;\mathrm{count}}\times 100$$

The Log reduction was calculated as follows:

Log10(initialcount)–Log10(×timeinterval)=Log10reduction

To obtain the time-kill curve, the bacterial strain growth rate was counted at different time intervals starting from 0 to 24 h, and these time intervals were plotted as a semi-log plot graph. The bacterial strains were plotted on the coordinate (*Y*-axis) on a logarithmic scale, while the corresponding time on the abscissa (*X* axis) on an arithmetic scale.

#### 2.2.9 Statistical analysis

Statistical analysis was performed using GraphPad Prism (version 10.6.1). All experiments were conducted in biological triplicates. Data were first tested for normality, followed by one-way ANOVA, with *post hoc* Bonferroni correction applied for multiple comparisons and *p*-value <0.05 was considered statistically significant.

## 3 Results

### 3.1 Physical characterization reveals conjugation of silk protein with MTA

**Figure 1:**
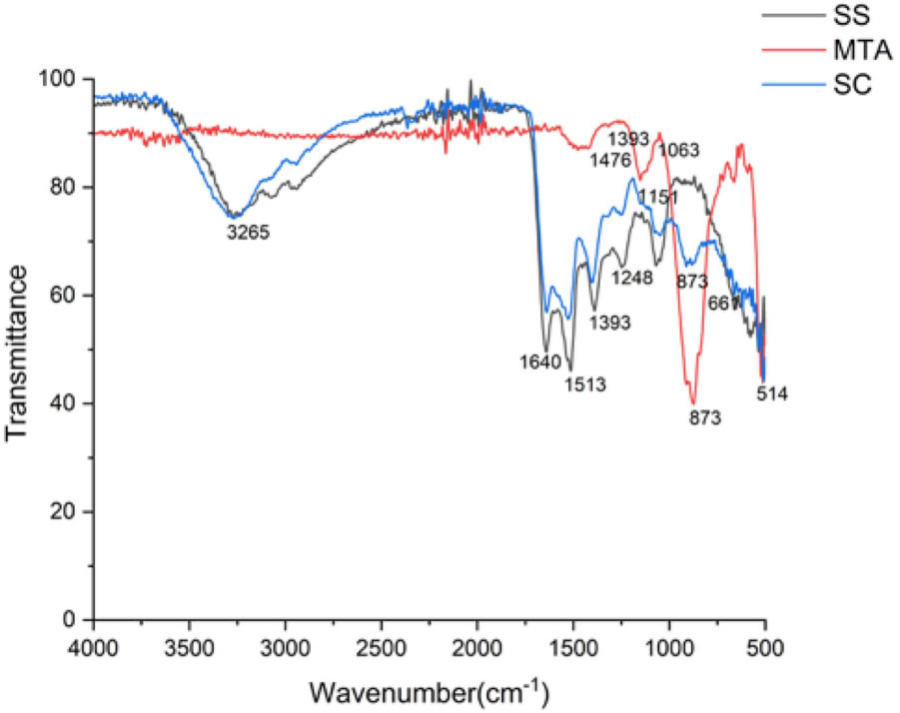
ATR- FTIR of silk sericin (SS), MTA, sericin-MTA conjugates (SC).

**Figure 2:**
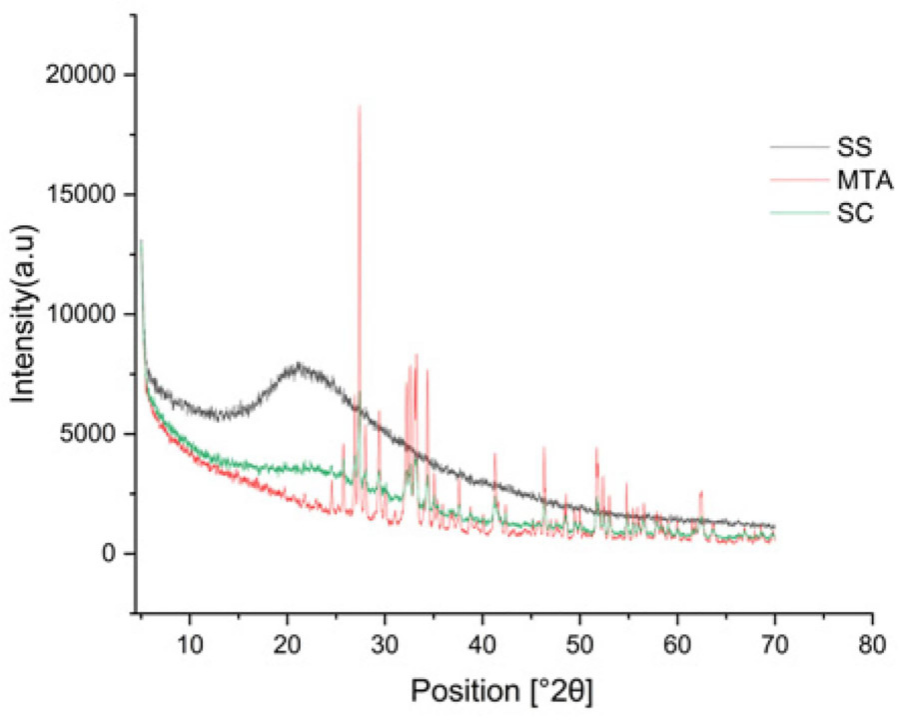
XRD pattern of silk sericin (SS), MTA, sericin-MTA conjugates (SC).

ATR-FTIR spectra confirm Sericin characteristic amide I(C = O stretching at ∼1,640 cm^−1^), amide II (N–H bending at ∼1,513 cm^−1^) and N–H stretching (3,265 cm^−1^), indicating its proteinaceous nature. MTA exhibits distinct carbonate (1,476, 1,393 cm^−1^), sulphate (1,151, 1,063 cm^−1^), and silicate (873,514 cm^−1^), confirming its inorganic composition. The sericin composite retains both amide and inorganic peaks, demonstrating successful integration while preserving sericin's functional groups. The presence of dicalcium silicate (873 cm^−1^) and calcium hydroxide bands suggests hydration with a noticeable water-associated peak at 661 cm^−1^. Despite hydration, the composite remains chemically stable, maintaining its structural integrity and functional properties. ATR-FTIR analysis confirmed the conjugation of silk protein with MTA (Figure 1). In addition to this, all the materials exhibited a lattice structure in the XRD analysis and were found to be crystalline in nature, having a size <100 nm. MTA showed large peaks representing bismuth oxide, calcium silicate oxide, and calcite were observed at 27.39°, 33.22 °, and 29.40 °, respectively. Peaks at 46.58° and 52.14° represent tricalcium aluminate. There are no noticeable differences in the composition and crystalline structure between the MTA and Sericin-MTA conjugate (Figure 2).

### 3.2 Sericin-MTA conjugate showed irregular surface characteristics

Silk sericin showed flat uniform base layer with uneven projections (Figure 3a). MTA appeared to have flat and uniform base layers that were filled with submicron-sized pits and nano-scale projections, evenly distributed across their surfaces (Figure 3b). Sericin-MTA conjugate showed shallow depressions across their surface with bulbous structure and irregular surface roughness measurements that were in the μm scale (Figure 3c).

**Figure 3a:**
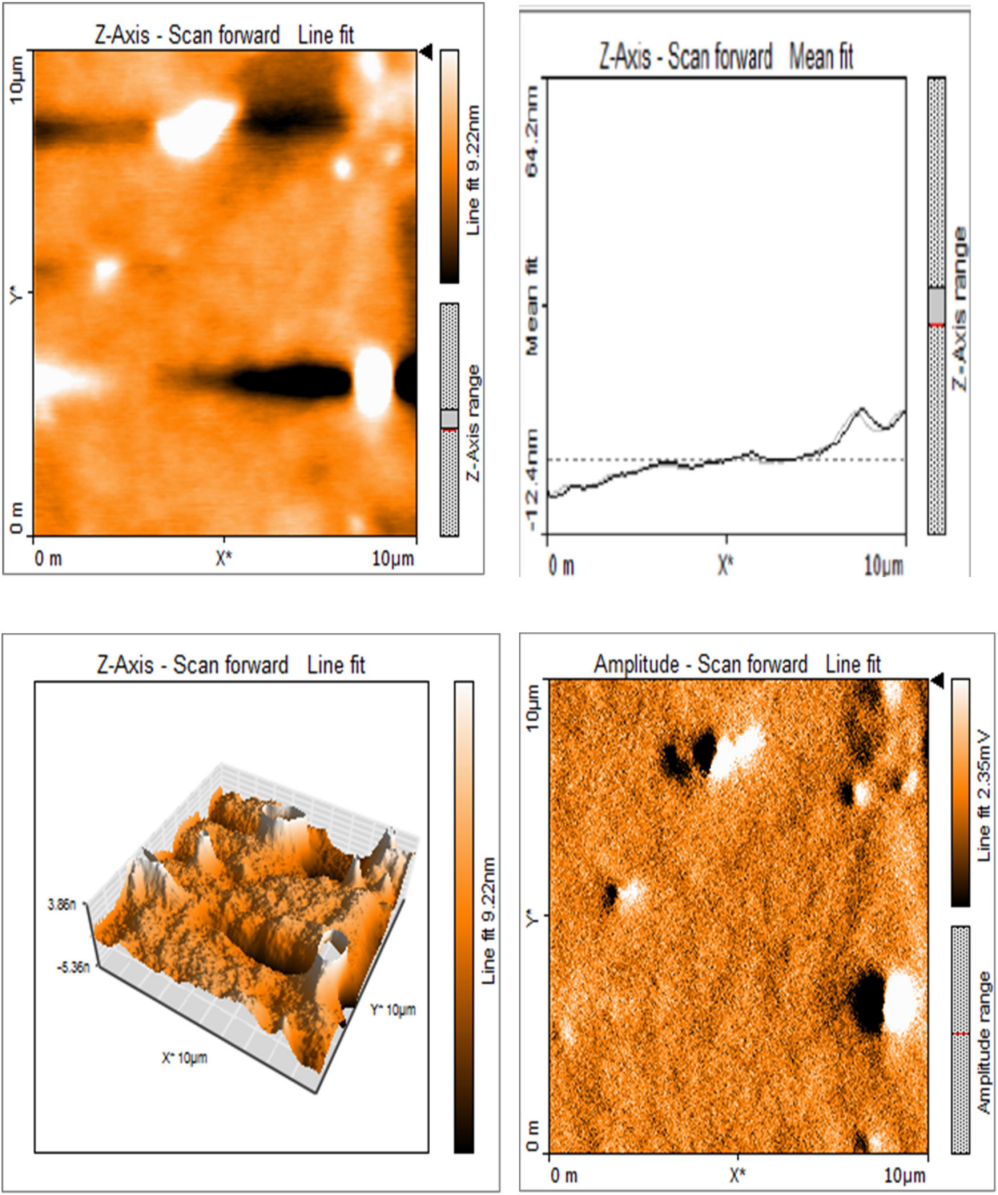
Representative images of samples using AFM: Topography and 3-D rendering of Silk Sericin showed flat uniform base layer with uneven projections.

**Figure 3b:**
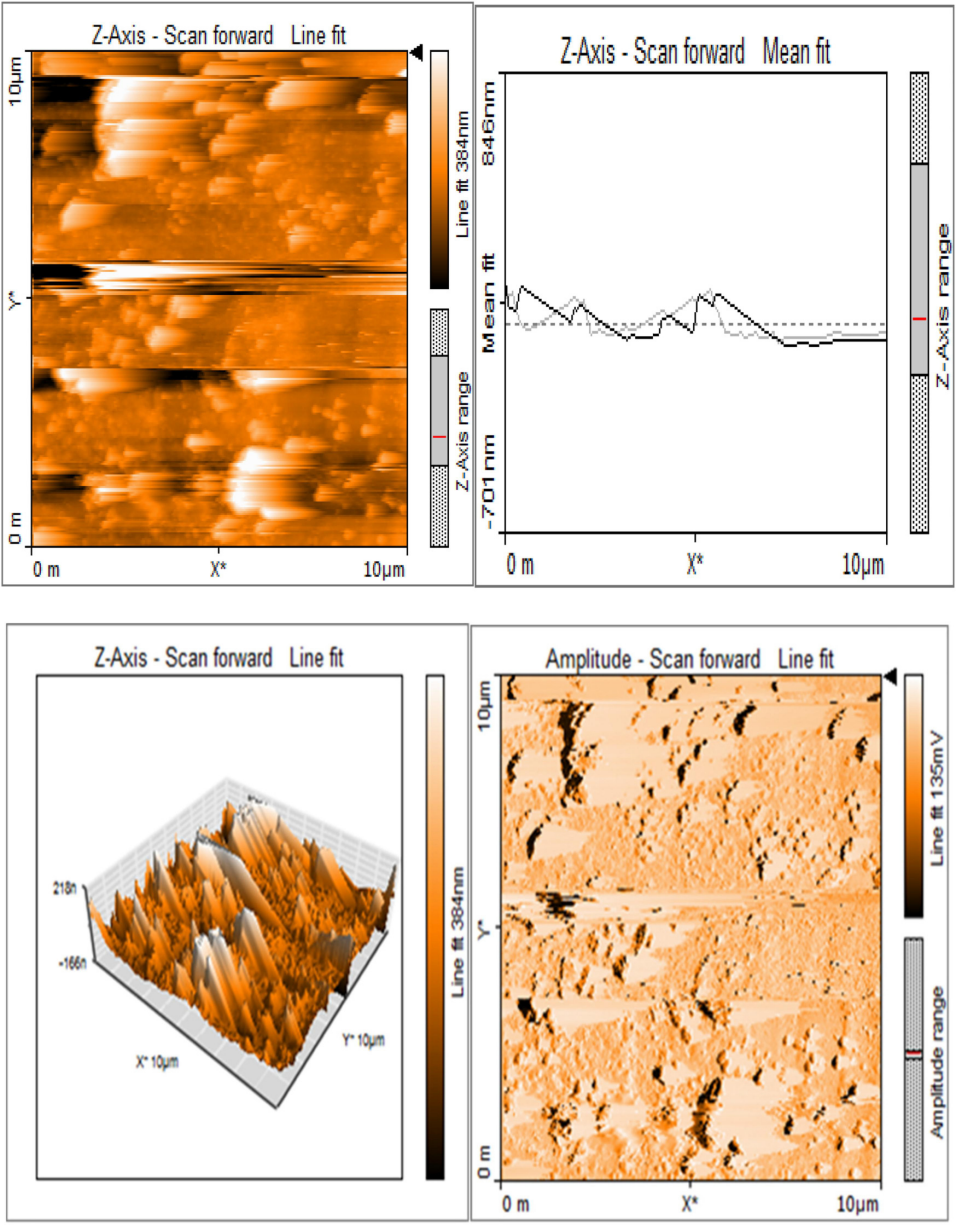
Base layers of MTA exhibiting a consistent and even texture, featuring submicron-sized pits and nano-scale projections uniformly distributed across their surfaces.

**Figure 3c:**
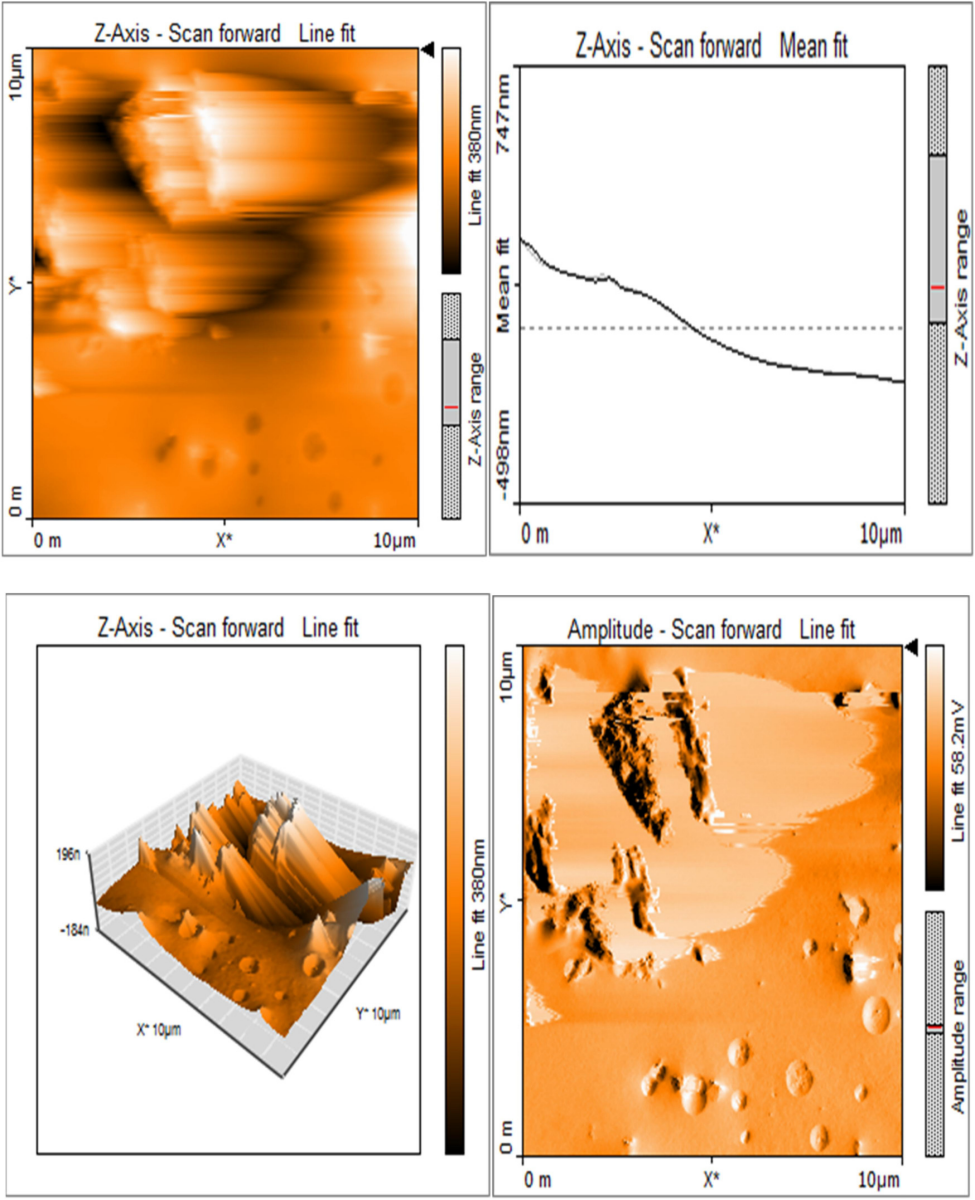
Sericin-MTA conjugate showing shallow depressions and a bulging, uneven surface.

### 3.3 Sericin-MTA conjugate showed helical structures in circular dichroism spectra analysis

**Figure 4:**
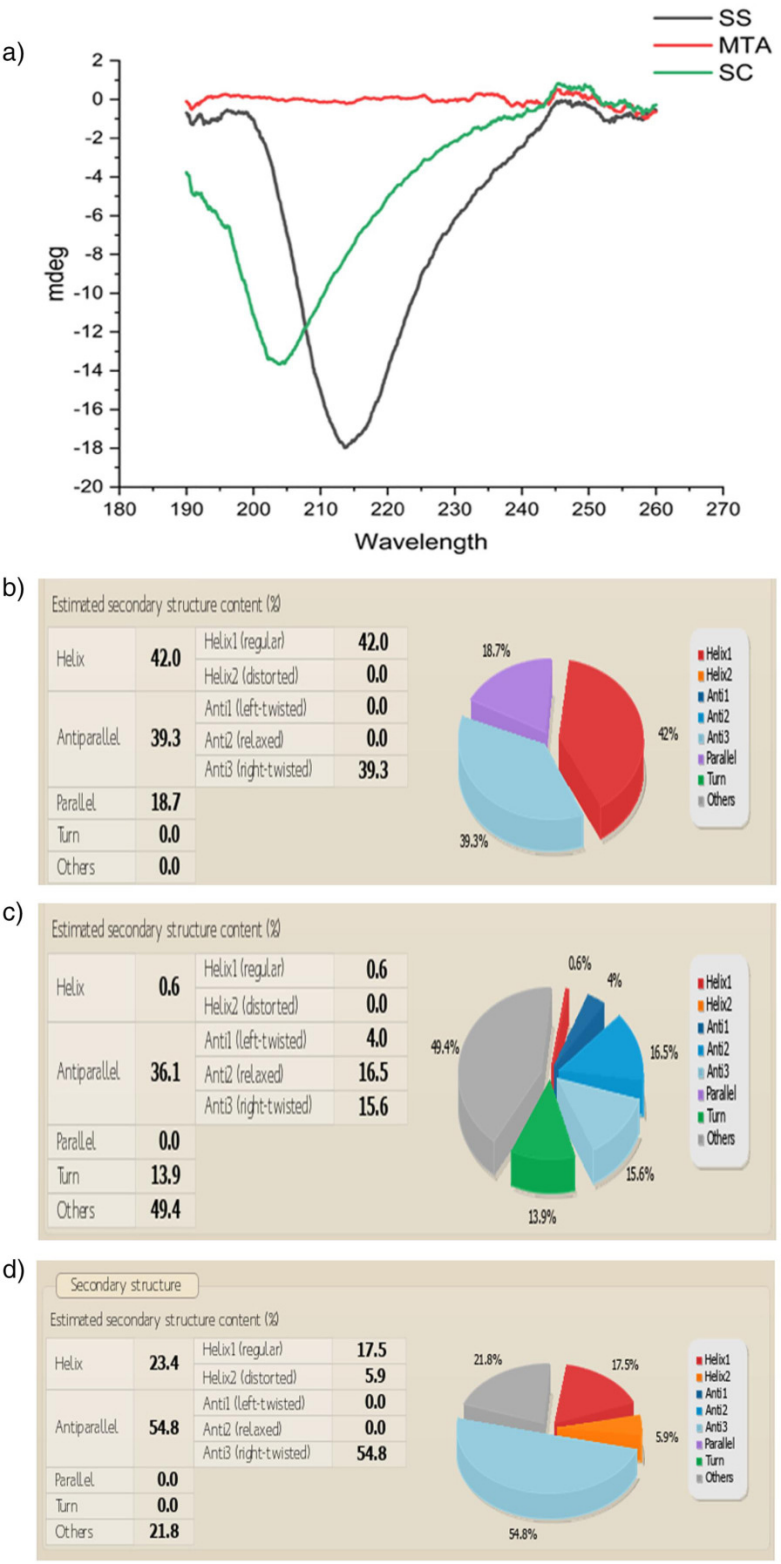
**(a)** Circular dichroism CD Spectra of a) Silk sericin (SS), silk sericin-MTA conjugate (SC). **(b)** CD spectral analysis of structural variations in Silk Sericin: Silk sericin reveals a structured secondary composition with 42% α-helix (Helix1), 39.3% anti-parallel β-sheet (Anti3), and 18.7% parallel β-sheet, while turns and unordered structures are absent (0.0%). The high β-sheet content suggests structural stability, typical of silk proteins, whileα-helix presence contributes to flexibility. **(c)** CD spectral analysis of MTA: MTA reveals a predominantly unordered structure (49.4%), followed by 36.1% antiparallel β-sheets (16.5% relaxed, 15.6% right twisted, 4.0% left twisted, 13.9% turns, and a minimal 0.6% α-helix. The absence of parallel β-sheets suggests a lack of strong structural rigidity, while the high disordered content indicates an amorphous nature. **(d)** CD spectral analysis of Sericin/MTA Conjugate (SC): Sericin/MTA conjugate shows a dominant antiparallel β-sheet structure (54.8%), primarily right-twisted (54.8%), indicating a strong silk-like structure typical of Sericin. The Helix content (23.4%) includes regular α-helices (17.5%) and distorted helices (5.9%), reflecting some ordered helical regions. There is no significant presence of left-twisted β-sheets (0.0%) or parallel β-sheets (0.0%), and no turns are observed in structures (21.8%), indicating a degree of flexibility or disordered regions in the conjugate. This secondary structure distribution highlights a balance between ordered β-sheets and helical elements, making the sericin-MTA conjugate suitable for a structurally stable.

Circular Dichroism (CD) Spectra Analysis reveals the distinctive helical structures observed in Silk sericin protein (SS) and conjugate (SC), validating the inherent protein characteristics. In contrast, the mineral compound MTA lacks noteworthy helical structures. (Figures 4a–d).

### 3.4 Sericin-MTA conjugate demonstrated antimicrobial activity

**Figure 5:**
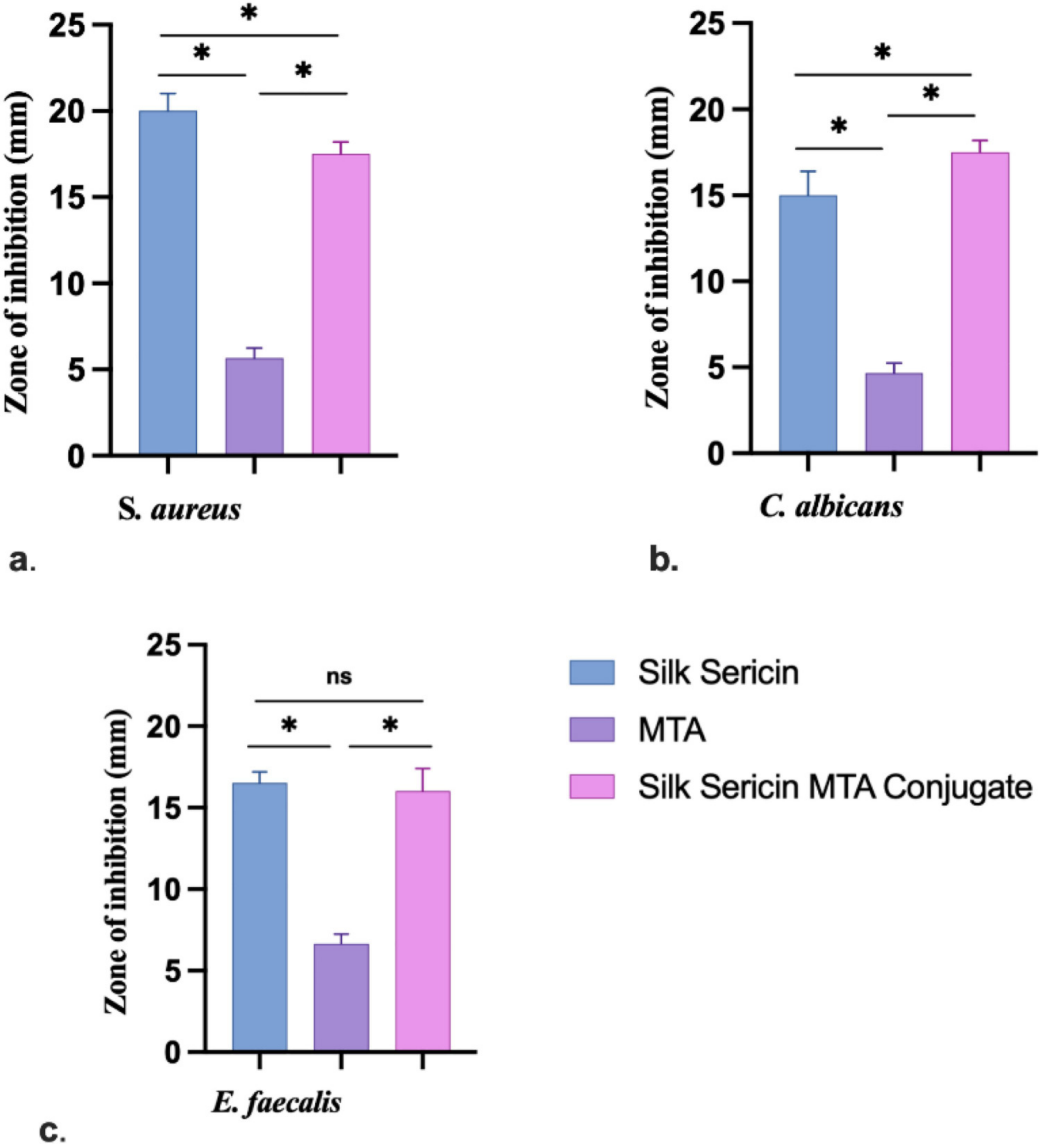
Zone of inhibition for antimicrobial activity: **(a)** *S. aureus*, **(b)** *C. albicans* and **(c)** *E. faecalis* (Data represented in mean ± SD, *n* = 3, *p < 0.05).

The samples sericin, MTA, and SC showed antimicrobial activity against all the test microorganisms. The details of antimicrobial activity are presented in Figure 5. Sericin samples and SC demonstrated marked antimicrobial activity, followed by MTA samples.

### 3.5 Sericin-MTA conjugate exhibited significant MIC at lower concentrations

**Figure 6:**
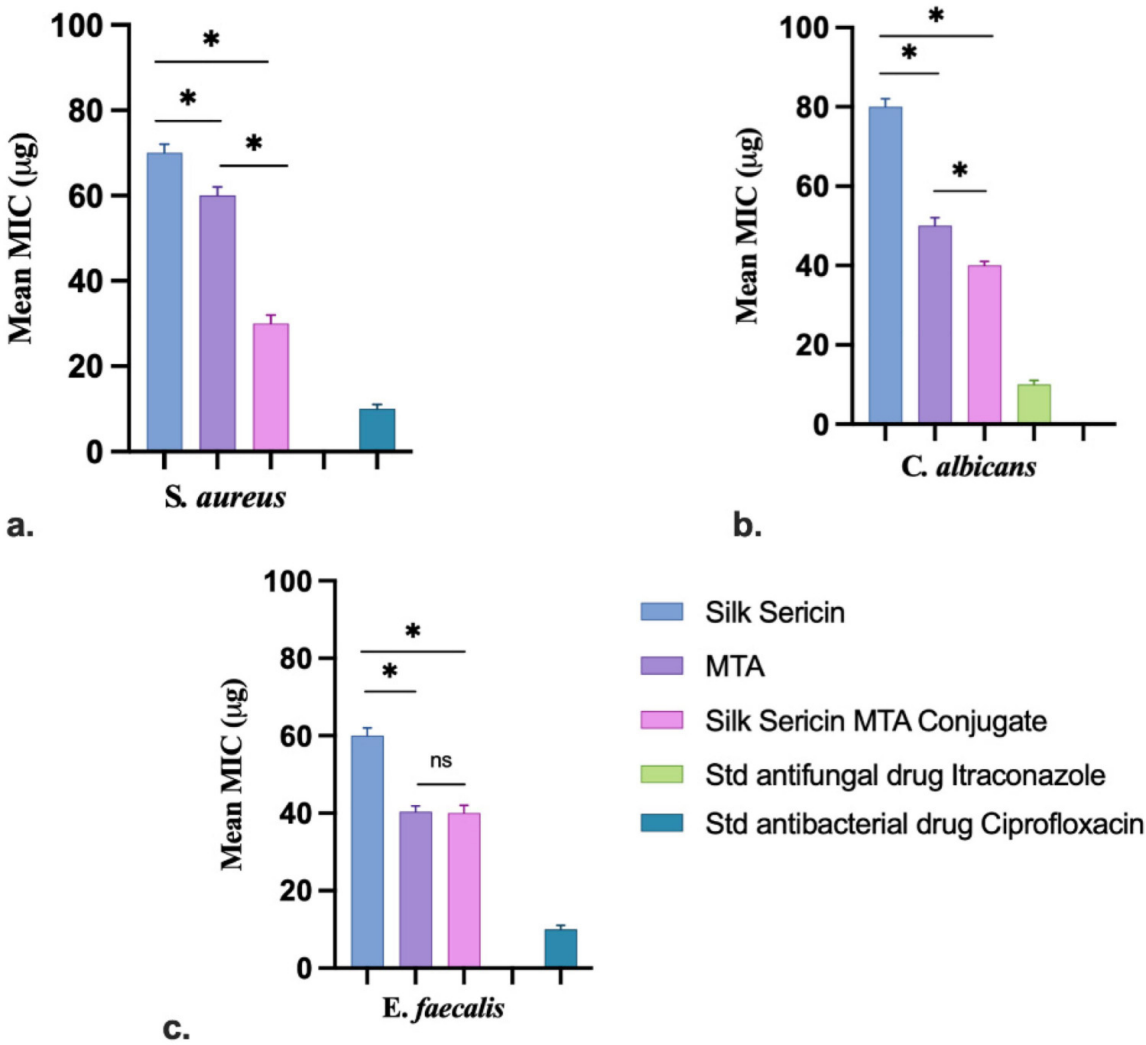
MIC test samples against different microbial pathogens: **(a)** *S. aureus*, **(b)** *C. albicans* and **(c)** *E. faecalis* (Data represented in mean ± SD, *n* = 3, *p < 0.05).

The Sericin-MTA conjugate samples have exhibited significant results at lower concentrations, followed by MTA and then Sericin samples. The detail of the Minimum Inhibitory Concentration (MIC) is presented in Figure 6.

The MIC for sample sericin for *E. faecalis*, *S. aureus,* and *C. albicans* was found to be 60 µg, 70 µg, and 80 µg, respectively. For the sample MTA, MIC was calculated to be 40 µg, 60 µg, and 50 µg. Further, in the case of Sericin-conjugated MTA, the MIC for tested organisms was found to be 40 µg, 30 µg and 40 µg, respectively. Whereas for the standard drug, Itraconazole and Ciprofloxacin were found to be 10 µg, respectively. MTT-based MIC study of test samples Sericin, MTA, and sericin-conjugated MTA were tested against *Enterococcus faecalis*, *Staphylococcus aureus,* and *Candida albicans* at different concentrations (10 µg–100 µg). The conjugated sample has shown prominent activity at a lower concentration 10 µg, for *Enterococcus faecalis*, *Staphylococcus aureus,* and for Candida albicans, it was at 20 µg. Whereas MTA has shown at 20 µg for *Enterococcus faecalis* and 10 µg for *Staphylococcus aureus,* and for *Candida albicans* it was at 30 µg. Further, the sample Sericin for *Enterococcus faecalis*, *Staphylococcus aureus,* and *Candida albicans* have shown for 70 µg, 10 µg, and 80 µg, respectively. Overall, the MTA-conjugated Sericin sample has exhibited significant antimicrobial activity. The results of MIC using the MTT Assay are presented in Table 1. In the MTT-based MIC study, the result was expressed in terms of resistance (*R*) and sensitive (*S*). The resistance means there was observation of the growth of selected organisms, whereas sensitive means there was no turbidity nor growth of the organisms.

**Table 1:** The results of Minimum inhibitory concentration using MTT assay.

Microorganisms	Test samples	Minimal inhibitory concentration (µg/ml)									
		10	20	30	40	50	60	70	80	90	100
*Enterococcus faecalis*	Silk Sericin	R	R	R	R	R	R	S	S	S	S
	MTA	R	S	S	S	S	S	S	S	S	S
	Silk Sericin Conjugate	S	S	S	S	S	S	S	S	R	R
*Staphylococcus aureus*	Silk Sericin	S	S	S	S	S	S	S	S	S	S
	MTA	S	S	S	S	S	S	S	S	S	S
	Silk Sericin Conjugate	S	S	S	S	S	S	S	S	S	S
*Candida albicans*	Silk Sericin	R	R	R	R	R	R	R	S	S	S
	MTA	R	R	S	S	S	S	S	S	S	S
	Silk Sericin Conjugate	R	S	S	S	S	S	S	S	S	S

R, resistant; S, sensitive.

### 3.6 Time-kill (antimicrobial kinetic) assay

**Figure 7:**
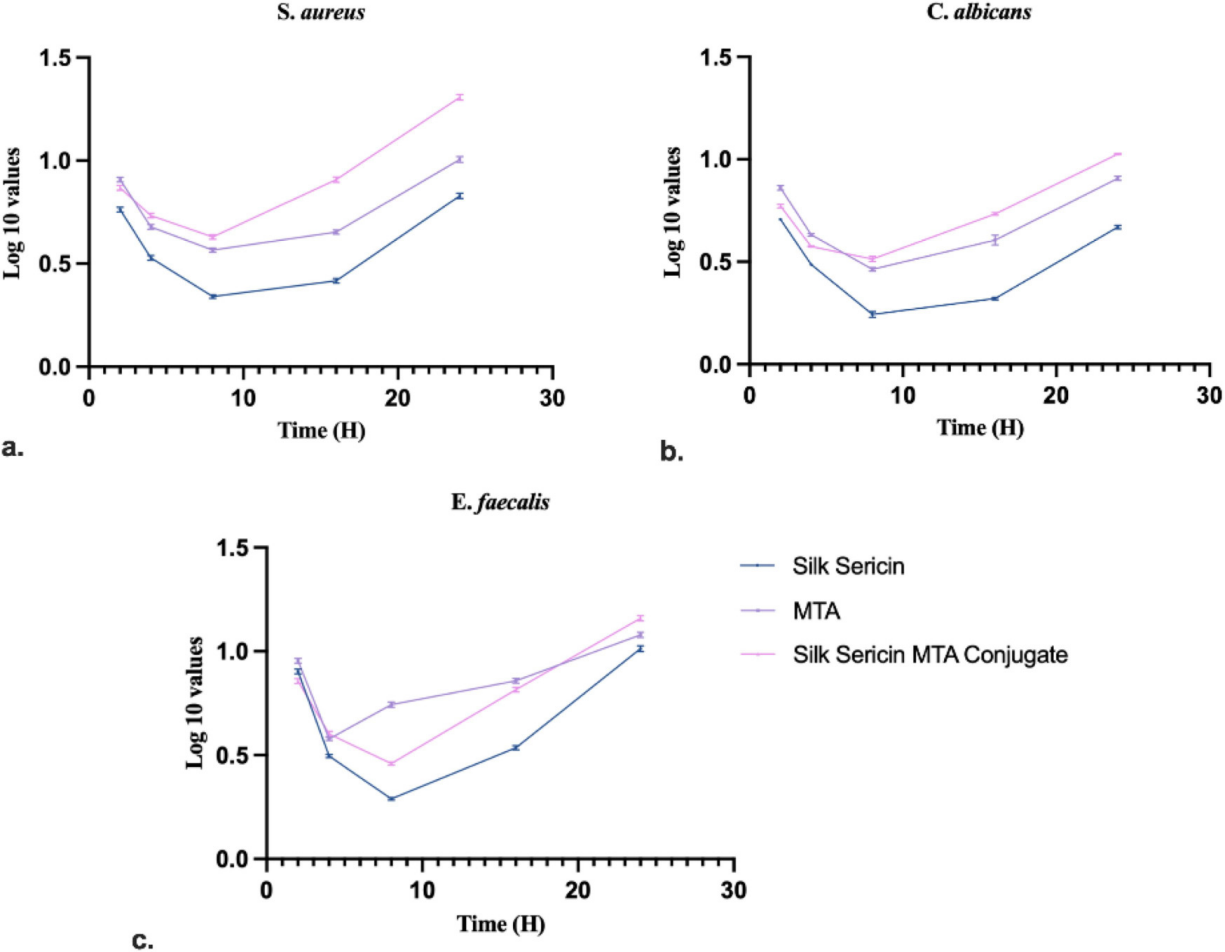
Time kill assay of test samples against: **(a)** *S. aureus*, **(b)** *C. albicans* and **(c)** *E. faecalis* (Data represented in mean ± SD, *n* = 3).

The time-kill method is used to test the bactericidal activity of one or more antimicrobial agents against a particular bacterial strain. The experimental outcome for *E. faecalis*, *S. aureus*, and *C. albicans* (Figure 7). The microbial load decreased significantly from the initial values, reaching the lowest point, followed by regrowth in all the test groups. Among the three groups, Silk Sericin demonstrated the most pronounced reduction in microbial count, while MTA and SC showed moderate inhibition.

## 4 Discussion

The findings of the present study demonstrated the successful incorporation of the silk-derived sericin into MTA. The biophysical characterization provides strong evidence supporting the structural stability and potential antimicrobial effects of the silk sericin-MTA composite.

ATR-FTIR confirmed the presence of key functional groups in all four samples: silk sericin (SS), Mineral Trioxide Aggregate (MTA), and the silk sericin-MTA conjugate (SC). The characteristic peaks of silk sericin (N–H stretching at 3,500–3,000 cm^−1^ and C=O stretching at 1,700–1,600 cm^−1^) were observed in all samples containing silk sericin (SS and SC), aligning with previous findings on the application of silk sericin to polyester fabric (38). This confirms the successful incorporation of silk sericin into the MTA composites. Additionally, characteristic peaks for calcium hydroxide, carbonate, silicate, and sulfate in the MTA-containing samples (MTA and SC) were consistent with expectations and previous studies on the bioactivity of MTA (39), which observed similar carbonate and silicate peaks. The similarity in functional group composition and peak positions of SC spectra suggests that the mixing method successfully retained the major components of the composite. The presence of a distinct peak at 661 cm^−1^ in the SC spectrum indicates the presence of water, suggesting that the silk sericin-MTA conjugate remains stable post-hydration, an essential feature for potential endodontic applications.

XRD analysis further reinforced these findings by demonstrating the crystalline nature of all four samples, with crystallite sizes under 100 nm. The distinct peaks for bismuth oxide, calcium silicate oxide, and calcite in the MTA sample are in line with the expected crystalline phases. Notably, the absence of significant differences in the crystalline structure between the MTA and SC samples indicates that silk sericin incorporation does not markedly alter the crystalline composition or structure. This retention of crystallinity in the sericin-MTA composite is significant as it suggests that the bioactive properties of MTA are preserved, which is crucial for clinical applications. The similarity in crystallinity with silk proteins was reported for fibroin, further supporting the stability of the material (40, 41).

Surface morphology analysis via AFM revealed significant topography differences upon silk-sericin incorporation. While MTA showed a relatively flat and uniform base layer with submicron-sized pits and nano-scale projections, the SC samples displayed shallow depressions and bulbous structures, indicating altered surface topography. Such surface modifications could enhance the biological properties of the material, such as cellular adhesion and protein absorption, which are crucial in facilitating tissue regeneration and wound healing. Similar morphological alterations have been observed in studies on silk sericin incorporation into other materials, such as the bacterial cellulose membrane, as demonstrated by Wang et al. (42). These surface changes could potentially influence the material's interaction with biological tissues, an important consideration for its intended biomedical use.

The Circular Dichroism spectra highlighted the preservation of the α-helical structure in silk sericin, even after its regeneration into powder form. This retention of the α-helical conformation in SC composites suggests that the incorporation of MTA does not significantly alter the secondary structure of silk sericin. This finding is consistent with previous work by Gulrajani et al., who also observed α-helix bands in sericin samples (43). The stability of the α-helical structure within these composites is an important characteristic, as this conformation is associated with advantageous mechanical and biological properties. The fact that MTA, as a mineral, does not exhibit significant helical features further underscores the importance of maintaining sericin's secondary structure. This structural stability enhances the potential of these composites for clinical applications, particularly in endodontics, where material bioactivity and structural integrity are of key importance. The antimicrobial evaluation of the samples revealed that silk-derived sericin significantly enhanced the antimicrobial properties of MTA against common oral pathogens. The well diffusion method indicated that the antimicrobial efficacy followed the order: Sericin-MTA conjugate < sericin with MTA alone, demonstrating the least inhibition. The innate antimicrobial properties of sericin are likely responsible for these enhanced antimicrobial effects in the sericin-MTA composite. The emergence of antibiotic-resistant strains of bacteria and fungi has become a significant public health concern, underscoring the need for the development of novel antimicrobial agents. Determining the Minimum Inhibitory Concentration of antimicrobial agents is a crucial step in evaluating their effectiveness against target microorganisms. However, the variability in methods used to determine the Minimal Lethal Concentration has been a concern, with differences in culture conditions, initial inoculum concentrations, and sampling protocols contributing to inconsistent results. The Microtiter Tetrazolium Assay has emerged as a promising technique for determining minimum inhibitory concentration. This method relies on the reduction of a tetrazolium salt by metabolically active cells, resulting in a colored formazan product that can be quantified using spectrophotometric analysis. The assay allows for the rapid and efficient screening of multiple antimicrobial agents and microbial strains simultaneously, making it a valuable tool for drug discovery and development. The alkalizing environment created by MTA plays an important role in inhibiting bacterial growth and enhancing the antimicrobial properties of MTA (44, 45). The superior antimicrobial efficacy of sericin samples could be attributed to their innate antimicrobial components, which might be effective in disrupting the bacterial and fungal cell structures. The regrowth observed after initial reduction in microbial load could be attributed to the depletion of antimicrobial components over time, as well as microbial adaptation and biofilm formation. These findings validate the hypothesis that conjugating sericin and MTA can create a synergistic effect, improving their efficacy against selected pathogens. Furthermore, the findings of the present study are in agreement with earlier reports for antimicrobial properties of MTA (46). The enhanced antimicrobial properties of Sericin-MTA conjugate could be due to the combined antimicrobial properties of sericin and MTA together. The reasons for differences in antimicrobial trends among assay types may be attributed to Silk 1 and Silk 2 components in sericin protein, while Silk 1 has antimicrobial properties and Silk 2 has antibacterial properties, especially for gram-negative bacteria (46). The bioactive silk protein, when used alone, its a surface-active nature that allows direct interaction with microbial cells and host tissues, maximizing its biological activity (29, 47). In contrast, when sericin is combined with MTA, the MTA matrix disperses sericin, reducing its immediate availability in soluble form for antimicrobial or bioactive action. Researchers have demonstrated that the hydration reaction of MTA leads to a dense calcium silicate hydrate structure that can encapsulate biomolecules, thereby slowing or limiting their release. This may attenuate the rapid biological response observed with sericin alone (shielding effect) (48). Similar kinds of interactions between organic biomolecules and inorganic cements have been reported in other composite systems as well, where entrapment within the cementitious matrix modulates release kinetics and can reduce the initial bioactivity (39, 49).

### 4.1 Limitations and future prospects

The incorporation of silk-derived sericin into MTA presents a promising avenue for improving the antimicrobial efficacy of biomaterials used in pulp therapies. The preservation of the innate crystalline structure of the MTA structure and the modified surface topography provides a unique opportunity to develop a bioactive material with enhanced antimicrobial effects. Further studies should focus on assessing these materials' mechanical properties and cytocompatibility on the cells of dental tissue origin and *in-vivo* animal trials. Additionally, optimizing the formula and developing a sustained/controlled release of the sericin's bioactive components could further improve the biological properties of the material.

## 5 Conclusions

Overall, the findings of the present study provide a strong foundation for the formulation of novel MTA-based biomaterials by conjugating silk-derived sericin. A significant increase in antimicrobial properties adds to the material's advantage. In the field of biomaterials, mechanical properties and biological properties, and antimicrobial nature play a crucial role in determining the suitability of the material. Hence, the outcome of the study suggests that a silk and MTA combination could be a potential biomaterial in dentistry. However, further animal and clinical trials are required to substantiate this evidence and explore the biocompatibility and cytotoxicity of this material.

## Data Availability

The original contributions presented in the study are included in the article/Supplementary Material, further inquiries can be directed to the corresponding authors.
